# Bio-Inspired Extreme Wetting Surfaces for Biomedical Applications

**DOI:** 10.3390/ma9020116

**Published:** 2016-02-19

**Authors:** Sera Shin, Jungmok Seo, Heetak Han, Subin Kang, Hyunchul Kim, Taeyoon Lee

**Affiliations:** Nanobio Device Laboratory, School of Electrical and Electronic Engineering, Yonsei University, 50 Yonsei-ro, Seodaemun-Gu, Seoul 03722, Korea; serashin0105@gmail.com (S.S.); kamacoon@yonsei.ac.kr (H.H.); sbin3106@gmail.com (S.K.); hyunchulkim0311@gmail.com (H.K.)

**Keywords:** superhydrophobicity, extreme wetting surface, bio-inspired surface, bio-mimicking, surface engineering, biomedical engineering

## Abstract

Biological creatures with unique surface wettability have long served as a source of inspiration for scientists and engineers. More specifically, materials exhibiting extreme wetting properties, such as superhydrophilic and superhydrophobic surfaces, have attracted considerable attention because of their potential use in various applications, such as self-cleaning fabrics, anti-fog windows, anti-corrosive coatings, drag-reduction systems, and efficient water transportation. In particular, the engineering of surface wettability by manipulating chemical properties and structure opens emerging biomedical applications ranging from high-throughput cell culture platforms to biomedical devices. This review describes design and fabrication methods for artificial extreme wetting surfaces. Next, we introduce some of the newer and emerging biomedical applications using extreme wetting surfaces. Current challenges and future prospects of the surfaces for potential biomedical applications are also addressed.

## 1. Introduction

Living species have modified their organs to adapt to their habitat, producing diverse natural extreme wetting surfaces. These surfaces have received tremendous attention in multiple disciplines because of their numerous potential applications [1,2,3,4,5,6,7,8,9]. Based on the developments of nanotechnology, various surface functions such as the self-cleaning ability of lotus leaves [10], anisotropic wetting of rice leaves [11], water-collection behavior of desert beetles [12], and liquid repellency of pitcher plants [13] have been mimicked by engineering surface topology and chemistry. Recently, bio-inspired surfaces with extreme wettability have been extensively used in biomedical applications because their unique wetting property provides engineered cellular microenvironments and cell-substrate interactions, which cannot be achieved using conventional cell and tissue culture platforms [14,15,16,17,18,19]. Despite this high interest of the utilization of extreme wetting surfaces in biomedical applications, only a few reports have been published to understand their prospective roles and applications in the biomedical field [20,21].

**Figure 1:**
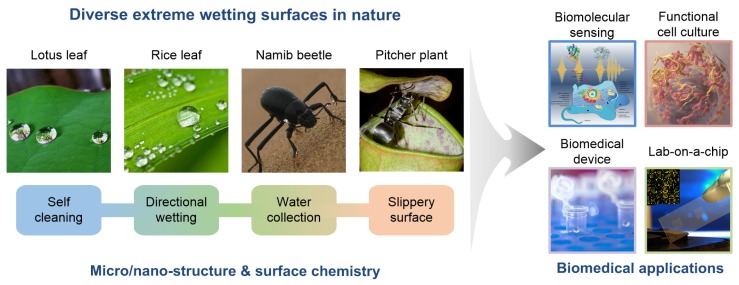
Various natural extreme wetting surfaces and their potential biomedical applications. Lotus leaf (image by Tanakawho, reproduced under Creative Commons Attribution (CC BY) license); Namib beetle (image by James Anderson, reproduced under Creative Commons Attribution Non-commercial Share-alike (CC BY-NC-SA) license); Pitcher plant (image by Bauer, reproduced under CC BY); Biomedical device (image from the School of Natural Resources & Environment, University of Michigan, reproduced under CC BY license); Lab-on-a-chip (image from Argonne National Laboratory, reproduced under CC BY-NC-SA license); and others (public domain photo and images).

In this review, we present natural extreme wetting surfaces and their emerging applications in the biomedical field (Figure 1). Section 2 introduces the design and fabrication of these surfaces inspired by the superhydrophobic water repellence of the lotus leaf, water adhesion of the rose petal, anisotropic wettability of the rice leaf and butterfly wing, patterned wettability of the desert beetle, and liquid slippery surfaces of the pitcher plant. Section 3 highlights emerging applications of extreme wetting surfaces to biomedical engineering, such as cell patterning for cellular interaction studies, functional cell spheroid cultures, biomedical devices, open-channel droplet-based lab-on-chips, and high-throughput cell assays. In addition to concluding remarks, this review discusses challenges and future prospects for the biomedical application of these surfaces.

## 2. Design and Fabrication of Bio-Inspired Extreme Wetting Surfaces

The unique surface wettability of certain living organisms has prompted scientists and engineers to design surfaces that mimic natural properties. Various artificial superhydrophobic surfaces have been fabricated as a result of advances in nanotechnology. Their functions, such as liquid repellence and anisotropic wettability, are achieved through an elaborate engineering of micro/nanostructures and surface energy. In this section, natural species exhibiting special wetting properties are introduced along with fabrication strategies for mimicking their functions.

### 2.1. Non-Wettable Superhydrophobic Surfaces

For the last decade, superhydrophobic surfaces showing a water contact angle (CA) exceeding 150° have received considerable interest because of their potential applications such as self-cleaning fabrics [6,22,23], no-loss droplet manipulation [24], and anti-corrosive coatings [8,25,26,27,28]. The researchers identified that non-wetting behavior of water droplets on superhydrophobic surfaces is governed by combination of surface chemistry and topology. In particular, superhydrophobicity could be achieved on nanostructured surfaces presenting a low surface energy. In general, these surfaces are described by Wenzel [29] and Cassie-Baxter models [30].

**Figure 2:**
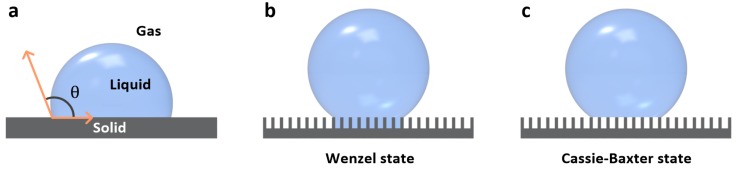
Schematic representations of a water droplet on flat and rough solid surfaces. (**a**) A droplet on an ideal flat surface; (**b**) A droplet in the Wenzel state, in which the rough surface is fully wetted; (**c**) A droplet in the Cassie-Baxter state, in which air pockets form at the interface between the non-wetted rough surface and droplet.

On an ideal flat solid surface (Figure 2a), a droplet forms a static CA (*θ*) with the surface. This CA is related to the surface energies of solid/gas (γ_sg_), solid/liquid (γ_sl_), and liquid/gas interfaces (γ_lg_) in Young’s equation [31]:

γ_sg_ = γ_sl_ + γ_lg_ × cos*θ* (1)

In case of a rough solid surface, effective values of γ_sg_, γ_sl_, and γ_lg_ should be considered because the actual contact area between the droplet and rough surface differs from the flat surface area. In the Wenzel model, it is assumed that the entire rough surface is in contact with the water droplet (Figure 2b). Here, water CA in Wenzel state (*θ*_w_*) can be determined using

cos*θ*_w_^*^ = *R* × cos*θ* (2)

where the surface roughness factor (R) corresponds to the ratio between the actual and projected surface areas. In this model, the water droplet is pinned onto superhydrophobic surfaces because of its large contact area with the surface. In the Cassie-Baxter model, it contacts the top-most layer of the rough surface, giving rise to air pockets at the liquid-solid interface (Figure 2c). Consequently, Young’s equation is expressed as

cos*θ*_c_^*^ = −1 + *f* (cos*θ* + 1) (3)

where θ_c_* is the Cassie-Baxter CA; and f is the ratio between the actual droplet contact area and the total surface area. This minimized liquid-surface contact area results in superhydrophobic surfaces exhibiting self-cleaning and water-repellent characteristics.

**Figure 3:**
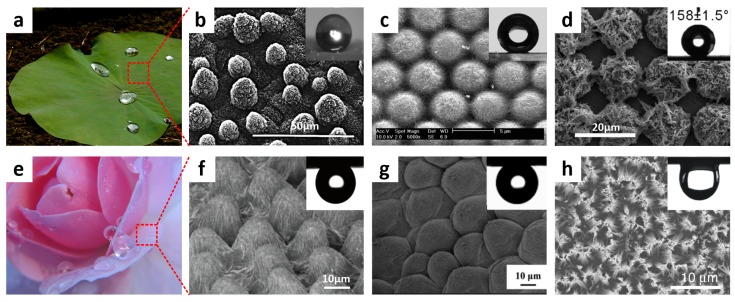
(**a**) Image of a lotus leaf (image by GJ Bulte, reproduced under Creative Commons Attribution Share-alike (CC BY-SA) license); (**b**) the corresponding scanning electron microscopy (SEM) image showing the hierarchical micro/nanostructures comprising papillose cells; (**c**) SEM image of a microsphere/single-walled carbon nanotube (CNT) composite array; (**d**) SEM image of a tetragonal array comprising Cu microprotrusions covered by nanostructured Ag dendrites; (**e**) Photograph of rose petals exhibiting water-adhesive properties; and (**f**) SEM image of rose petal surface (image by Audrey, reproduced under CC BY license); (**g**) SEM image of a rose petal-like polystyrene (PS)-film, onto which a water droplet was pinned even when turned upside down; (**h**) SEM image of Si nanowire arrays; Inset: water droplet deposited on the array after rapid thermal annealing (RTA) at tilt angle of 180°. Reproduced from [32,34,61,62,63,64] with the permission by Springer, Copyright 1997 and by ACS Publications, Copyright 2007, 2008, 2013 and by Elsevier, Copyright 2013.

Lotus leaves epitomize water-repellent superhydrophobic surfaces in nature (Figure 3a). In 1997, Barthlott and Neinhuis revealed for the first time that the lotus leaf has micro/nano hierarchical structures comprised of randomly oriented papillose epidermal cells covered with hydrophobic epicuticular wax (Figure 3b) [10,32]. They also observed that air can be trapped under a floating water droplet, consistent with the Cassie-Baxter equation. These findings suggested that lotus leaf-like highly water-repellent superhydrophobic surfaces can be fabricated using micro/nano hierarchical structures coated with low-surface-energy materials. Inspired by the lotus leaves, various methods such as wet chemical etching [33,34,35,36,37,38], electrochemical reaction [39,40,41], lithography [42,43,44], electrodynamics [45,46,47], sol-gel methods [48,49,50,51,52], layer-by-layer deposition [53,54,55], and plasma treatment [56,57,58,59] have been developed to produce these surfaces. Jiang *et al.* reported an electrohydrodynamics technique as a versatile and effective method to fabricate polystyrene (PS) composite film combining porous microspheres and inter-woven nanofibers [60]. The fabricated PS composite film showed superhydrophobicity owing to the increased surface roughness attributed to porous microspheres and inter-woven nanofibers. Li *et al.* developed hierarchical structures comprising periodically ordered PS colloidal crystals decorated with carbon nanotubes (CNTs) using a wet chemical self-assembly coating method (Figure 3c) [61]. The resulting surfaces exhibited superhydrophobicity with a low sliding angle (SA) after surface modification with 1H,1H,2H,2H-perfluorodecyltrichlorosilane (PFDTS). Recently, Zhang *et al.* fabricated hierarchical micro/nanostructure regular array comprising microprotrusion structured Cu covered by nanostructured Ag dendrites via electrochemical reactions combined with photolithography. These fabricated surfaces showed self-cleaning properties across a wide pH range after PFDTS modification (Figure 3d) [62].

In nature, species that have water pinning characteristics with superhydrophobicity have also been observed. For instance, a rose petal shows a high water CA with water-adhesive property. A droplet on rose petal does not roll off even when the surface is tilted vertically or turned upside down (Figure 3e). A periodic array of micropapillae and nanoscale cuticular folds on top of each micropapillae were observed on the surface of the rose petal (Figure 3f) [63,65]. This relatively large periodic array exerts a capillary force that facilitates water droplet penetration into micropillae grooves, explaining the water adhesive property. To mimic the liquid adhesion function of the rose petal, Feng *et al.* replicated the rose petal’s micro/nanostructures using the solvent-evaporation-driven nanoimprint pattern transfer process [63]. In the duplication process, a poly(vinyl alcohol) (PVA) film was negatively replicated to provide a second template. The PS film obtained from this negative replica exhibited water-adhesive superhydrophobicity with a large water CA (Figure 3g). Lai *et al.* reported nanostructured superhydrophobic TiO_2_ films with tunable surface adhesions by changing the diameter and length of nanotubes through a simple electrochemical method [65]. Water-adhesive force of the superhydrophobic porous nanostructures was engineered by utilizing surface roughness and capillary force. Recently, Seo *et al.* presented vertically-aligned silicon nanowire (Si NW) surfaces, whose wettability could be controlled from superhydrophilic to superhydrophobic upon rapid thermal annealing (RTA) at 1000 °C under ambient oxygen (Figure 3h) [34]. After the RTA cycle, the surfaces showed a significant water CA change from 0 to 154° and high water-adhesive properties. This drastic transformation was mainly attributed to the surface chemistry change of hydrophilic silanol groups (-Si-O-H) into hydrophobic siloxane groups (-Si-O-Si-).

**Figure 4:**
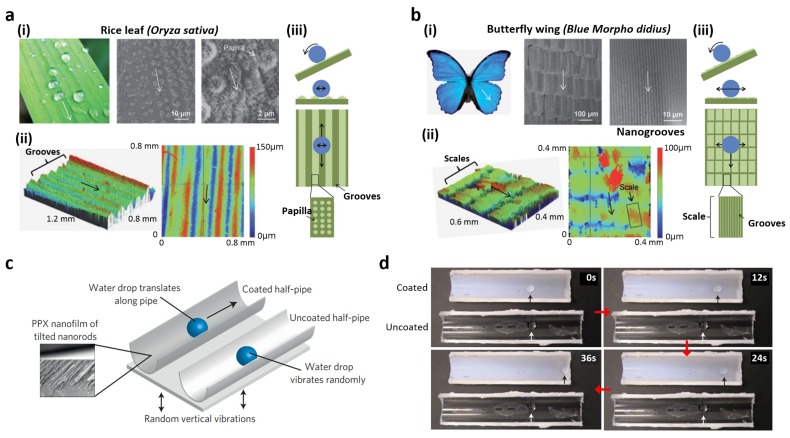
(**a**) Bidirectional anisotropic wetting of a rice leaf (*Oryza sativa*): (**i**) Photograph and SEM images of the rice leaf; (**ii**) Optical profiler height map of the rice leaf; (**iii**) Bidirectional anisotropic wetting behavior; (**b**) Unidirectional wetting behavior of a butterfly wing (*Blue Morpho didius*): (**i**) Photograph and SEM images of the Blue Morpho didius butterfly wing; (**ii**) optical profiler height map of the butterfly wing; and (**iii**) unidirectional anisotropic wetting behavior; (**c**) Droplet motion on poly(p-xylylene) film of tilted nanorods; and (**d**) corresponding time-lapse frames of droplet motion. Reproduced from [66,67] with the permission by Royal Society of Chemistry, Copyright 2012 and by Nature Publishing Group, Copyright 2010.

Apart from the superhydrophobicity of lotus leaves and rose petals, anisotropic wettability of natural species, which is exemplified by rice leaves and butterfly wings, has been studied. Similar to the lotus leaf, a rice leaf presents hierarchical structures covered with waxy nanobumps (Figure 4a(i)); however, a quasi-one-dimensional arrangement of the micropapillae leads to the anisotropic wettability (Figure 4a(ii)) [11]. This directional arrangement provides different energy barriers for wetting depending on the orientation, allowing a droplet to easily roll off perpendicular to the rice leaf edge (Figure 4a(iii)). To fabricate artificial superhydrophobic surfaces exhibiting anisotropic wetting property, several methods have been developed [11,68,69,70,71,72,73,74]. Wu *et al.* fabricated directionally aligned poly(vinyl butyral) nanofiber arrays by electrospinning [70]. In the electrospinning process, nanofibers are deposited between two parallel copper strips to generate uniaxially aligned arrays over large areas. A water droplet showed anisotropic wetting behavior on the aligned fiber arrays, similar to a rice leaf. By utilizing the fiber collector patterns, directional wetting behavior can be engineered. Recently, Kang *et al.* developed three types of anisotropic microgroove arrays presenting various shapes such as those of prism, rectangle, and overhang structures using UV-assisted micromolding process and subsequent surface modification with octafluorocyclobutane [75]. On the various microgrooves with different entry shapes, wetting behaviors on the surface such as static CAs and SAs were changed. In particular, overhang line arrays exhibited the highest liquid repellency and anisotropic wetting. The grooves guided water droplets as well as mineral oil droplets because of the extremely low surface energy of octafluorocyclobutane (~13 mJ/m^2^) and the overhang structure. 

In addition to anisotropic wetting, certain natural surfaces with unidirectional wetting have been observed in nature. The wings of the butterfly *Morpho* show unidirectional wetting properties because their surface presents shingle-like micro/nanostructures associated with aligned microgrooves (Figure 4b(i)) [66,76] These ratchet-like micro/nanostructures, which are periodically overlapped outward with respect to the wing (Figure 4b(ii)), allow a droplet to easily roll off the wing while being tightly pinned in the opposite direction (Figure 4b(iii)). To mimic the unidirectional wetting behavior of the butterfly wing, several anisotropic surfaces have been engineered. Recently, Malvadkar *et al.* investigated unidirectional wetting behavior on tilted nanofilm with poly(p-xylylene) (PPX) nanorod array [67]. The tilted PPX nanorods were fabricated by oblique angle polymerization, during which the vapor flux was directed at a controlled shallow angle to achieve shadowing and selective polymer growth. The resulting PPX nanorod film exhibited larger water CAs and unidirectional wettability. When the surface was oriented vertically with nanorods pointing upward, water droplets adhered to the surface. In contrast, these droplets rolled off the surface in the opposite direction. The unidirectional droplet movement was demonstrated by applying low-amplitude vibrations to half-pipe structures coated with the PPX film (Figure 4c). When random vibrations were applied to droplet located half-pipes, a unidirectional motion of the droplet was observed in the PPX nanofilm-coated half-pipe, but vibrated randomly on the uncoated half-pipe (Figure 4d). 

### 2.2. Patterned Wettabiltiy for Water Collection

**Figure 5:**
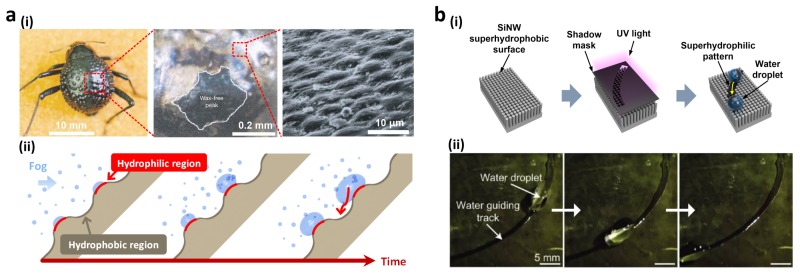
(**a**) Water-capturing surfaces of fused *Stenocara* beetle overwings: (**i**) Photograph and SEM images of wax-stained (colored) and unstained beetle wing regions (wax-free; black); (**ii**) Time-dependent growth of water droplets in a fog-laden wind; (**b**) Hydrophilic-patterned superhydrophobic Si nanowire (NW) arrays for water droplet guiding: (**i**) Fabrication of tilted Si NW arrays featuring a water guiding track; (**ii**) Sequential photographs of a water droplet guided along the hydrophilic track. Reproduced from [12,77] with permission by Nature Publishing Group, Copyright 2010 and by ACS Publications, Copyright 2011.

Biological organisms that live in extreme environments have adapted to survive under their environments. In particular, desert plants and animals exhibit specific features to get and conserve moisture in arid environments. Specifically, *Stenocara* beetles in the Namib Desert gather and condense water from fog on their bumpy back. This water-collation process relies on their hydrophilic and hydrophobic patterns existing on the back surface [12]. The wings of *Stenocara* are covered by a random array of hydrophilic smooth peaks and hydrophobic wax-coated rough troughs (Figure 5a(i)). Fog water settles on the hydrophilic peaks and condenses until they are entirely immersed. At a critical size, the droplet rolls down along the hydrophobic troughs (Figure 5a(ii)). 

Inspired by the hydrophilic patterned beetles’ backs, many wettability-patterned surfaces have been produced on various inorganic and organic materials such as metals, oxides, and polymers [78,79,80,81,82,83,84,85,86,87,88,89]. Recently, Seo *et al.* demonstrated hydrophilic-patterned superhydrophobic Si NW arrays to guide water droplets [77]. Superhydrophobic Si NW arrays were fabricated by metal-assisted electroless etching and self-assembled monolayer coating while hydrophilic guiding patterns were subsequently defined under UV illumination using shadow masks (Figure 5b(i)). Time-lapse photographs showed that a water droplet moved along the pre-defined hydrophilic tracks because of high wettability contrasts with surrounding superhydrophobic Si NW arrays (Figure 5b(ii)). Kang *et al.* presented a mask-free, solution-based chemical method based on adhesion mechanisms found in mussel adhesive proteins [82]. An artificial superhydrophobic porous oxide membrane surface was covered by patterned polydimethylsiloxane (PDMS) mold to partially coat mussel-inspired polydopamine (pDA). Because of its hydrophilicity, the patterned area became water adhesive and the surface could collect and guide water droplets on the pDA pattern. Similarly, Li *et al.* developed a superhydrophobic surface with printed superhydrophilic patterns [88]. A porous superhydrophobic surface was printed with phospholipid ink solution. The large difference in wettability between printed spots and a superhydrophobic surface, resulted in the selective wetting of the spot area by aqueous solutions. This printing technique offers a facile and simple approach to creating superhydrophilic patterns because of its compatibility with well-established microfabrication techniques, such as microcontact printing, inkjet printing, and dip-pen nanolithography.

### 2.3. Liquid Slippery Surfaces

**Figure 6:**
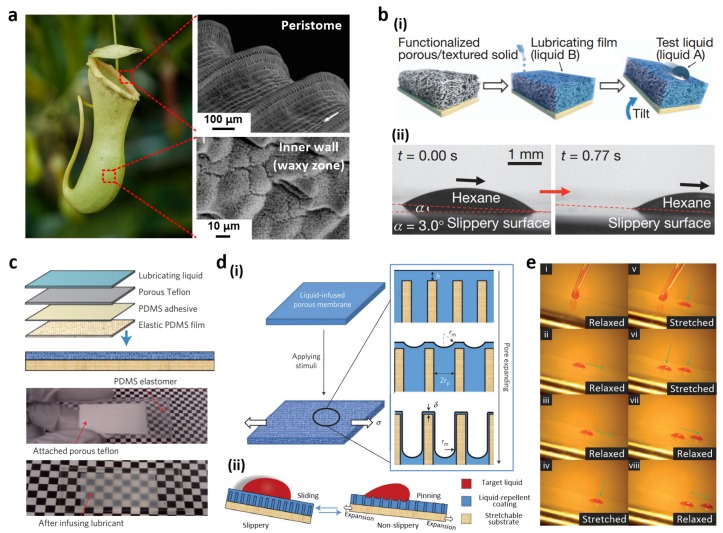
(**a**) Image of the *Nepenthes* pitcher plant (image by William Warby, reproduced under CC BY) and SEM images of the peristome surface and inner wall. The surface presents radial ridges, while the inner wall is covered with waxy crystals; (**b**) (**i**) Slippery film fabrication; (**ii**) Optical micrographs of a sliding hexane drop at a low angle; (**c**) Porous matrix formation on an elastic PDMS film and photographs of dry and lubricated substrates; (**d**) (**i**) Mechanically induced topographical changes in a liquid slippery film upon stretching and (**ii**) corresponding droplet motions; (**e**) Sequential photographs of oil droplet movement on the dynamic slippery surface. Reproduced from [13,93,94] with permission by National Academy of Sciences of the USA, Copyright 2004 and by Nature Publishing Group, Copyright 2011, 2013.

Most insects exhibit two functional features to attach to various surfaces. Claws facilitate clinging to rough surfaces, whereas adhesive pads promote sticking to smooth surfaces [90]. The tropical carnivorous pitcher plants *Nepenthes* benefits from a specialized trapping organ that impedes insect attachment. This organ mainly relies on the unique anisotropic and slippery characteristics of the peristome-pitcher rim (Figure 6a) [13,91,92]. The epidermal cells on the peristome have stacked microstructures toward the inside of the pitcher, covered with hydrophobic wax. In addition, the hydrophilic peristome surface promotes fluid film formation by secreted nectar and rain water. These physicochemical properties prevent claws from interlocking and adhesive pads from attaching to the peristome.

Inspired by the exceptional repellent ability of the *Nepenthes* pitcher plant, Wong *et al.* fabricated slippery surfaces by infiltrating liquid lubricants into nanostructured porous solids with a low surface energy (Figure 6b(i)) [93]. These slippery films were obtained using non-volatile perfluorinated lubricants immiscible with water- and oil-based solutions. Compared with non-infused, superhydrophobic surfaces, these films exhibited enhanced liquid repellent properties, showing negligible CA hysteresis and low SAs (Figure 6b(ii)); furthermore, these films had multiple functionalities such as instantaneous and repeatable self-healing, pressure stability, and optical transparency. They have been implemented in several applications such as marine anti-biofouling coatings [95], anti-icing [96], and anti-bacterials [97].

In addition to the aforementioned applications of slippery surfaces, mechanical stimulus-responsive slippery surfaces have been developed. Yao *et al.* fabricated a droplet adhesion controllable slippery surface on an elastic PDMS membrane (Figure 6c) [94]. The dynamic liquid interface reversibly switched between relaxed and stretched states (Figure 6d). In the relaxed state, the flat and smooth liquid interface promoted droplet sliding. In contrast, stretching exposed a rough surface that pinned the droplet (Figure 6e). In this stretched state, even a newly formed oil drop was immobilized while an existing one stayed at its deposition location. Both drops began to slide as soon as the stress was released. They also investigated the effect of different deformation mechanisms such as bending, poking, reversible swelling-drying, and self-healing.

### 2.4. Wettability Switchable Superhydrophobic Surfaces 

The reversible switching of superhydrophobic adhesion properties is greatly desired for no-loss transportation and biochemical detection requiring minimal liquid-substrate interaction is required. Stimuli-responsive materials offer new strategies to achieve this behavior on the same substrate [3,24,100,101,102,103,104,105,106,107,108,109]. Li *et al.* first reported the switching of superhydrophobic adhesion properties on a rough surface coated with a thermally responsive side-chain liquid crystal polymer (LCP) [102]. When the temperature surpassed a phase transition point, a water droplet was pinned onto the polymer-coated surface because polymer chains underwent a rearrangement from a hydrophobic to hydrophilic configuration. The reversibility of this adhesion was observed when the substrate was cooled down to room temperature. Moreover, temperature, pH, and electrolyte responsive polymers have been incorporated in rough surfaces to perform multi-responsive adhesion switching [103]. Photo-responsive materials have also been implemented to achieve adhesion switching surfaces. Wang *et al.* reported that superhydrophobic TiO_2_ nanotube films exhibited switchable water adhesion under UV irradiation and heat treatment [110]. These films were selectively illuminated through a mask to produce uniformly distributed hydrophilic regions in the superhydrophobic regions. These well-separated illuminated regions enhanced water adhesion, while preserving superhydrophobicity. Illuminated regions recovered their wettability through simple annealing processes (80–180 °C).

**Figure 7:**
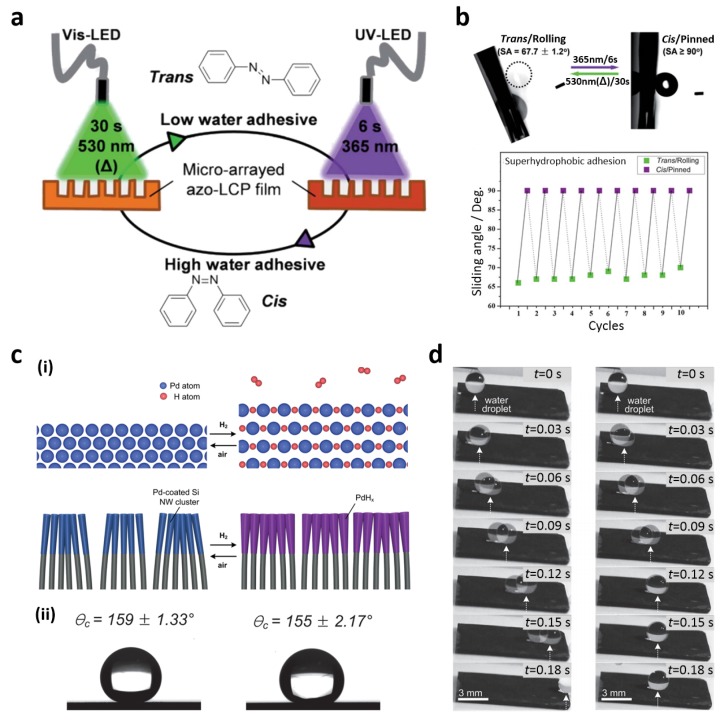
(**a**) Switchable adhesion under UV and visible light irradiation; (**b**) Reversible adhesion of superhydrophobic azobenzene liquid crystal polymer (LCP) film; (**c**) (**i**) Volume expansion of Pd layers deposited on the Si NW arrays under atmospheric and H_2_ ambient conditions; (**ii**) Contact angles (CAs) of the Pd-coated Si NW arrays showing superhydrophobicity under atmospheric and H_2_ conditions; (**d**) Time-lapse photographs of a moving water droplet on Pd-coated Si NW arrays under atmospheric and H_2_ conditions. Reproduced from [98,99] with the permission by Royal Society of Chemistry, Copyright 2012 and by John Wiley and Sons, Copyright 2013.

However, this strategy is unsuitable for practical applications because a temperature increase may dramatically accelerate the evaporation of water droplets. Unlike their temperature-responsive counterparts, photo-responsive materials benefit from localized stimulation and low thermal effect. Li *et al.* fabricated a photo-responsive rough surface using an azobenzene LCP showing a distinct polarity change between *trans* and *cis* isomers according to the irradiation wavelength [98]. Upon UV irradiation (365 nm, 6 s), azobenzene mesogens at the surface of fabricated azobenzene LCP film adopted a *cis* state and the concomitant polarity change enhanced the surface water adhesion. Upon visible light irradiation (530 nm, 30 s), azobenzene mesogens returned to their *trans* state and the surface retrieved its low water adhesion (Figure 7a). Consequently, the surface wettability was reversibly switched from rolling to the pinned state by alternating UV and visible light irradiations (Figure 7b). 

Because of their chemical nature, the above-mentioned strategies to control the superhydrophobic surface adhesion properties present several limitations regarding biological applications. For example, enzymes, biological cells, and chemicals would be affected by UV irradiation. As an alternative approach for tuning an adhesion force of a superhydrophobic surface, Wu *et al.* demonstrated that a superhydrophobic periodic PDMS micropillar array can be tuned by varying its curvature [24]. A water droplet adopted a pinned state on a flat PDMS pillar-array film with high adhesion force. Also, the adhesion gradually decreased with increasing surface curvature. When the curvature increased further, the droplet was detached from the array film. This phenomenon enabled *in situ* water droplet transportation without any loss. Recently, a new strategy using gas-responsive materials was developed to overcome the limitations of chemical techniques. A new strategy was also reported using gas-responsive materials to overcome limitations of the aforementioned techniques. Seo *et al.* demonstrated fast gas-driven adhesion switching of water droplets on a superhydrophobic Si NW array coated with a palladium (Pd) layer [99]. The quick adhesion switching of the Pd-coated Si NW arrays was achieved by a morphological phase transition of the coated Pd layer. Under H_2_ atmosphere, H atoms arising from H_2_ decomposition were incorporated into the as-deposited Pd layer, causing its volume to expand (Figure 7c(i)). When the ambient conditions switched from H_2_ to atmosphere, the expanded Pd layer instantly contracted to its initial volume. These Pd-coated Si NW arrays exhibited superhydrophobicity under air and H_2_ atmospheres (Figure 7c(ii)); however, they presented low and high adhesion properties under air and H_2_ atmospheres, respectively (Figure 7d). Droplet rolling off and pinning were also achieved repeatedly by alternating between these atmospheric and H_2_ conditions.

Section 2 summarized the design and fabrication of bio-inspired superhydrophobic surfaces with special wetting properties. Surface micro/nanostructures and energies provided elaborate control over wetting behaviors and liquid adhesion. These unique wetting properties of bio-inspired surfaces have the potential to be utilized in various biomedical applications, which cannot be achieved via conventional methods using flat petri dishes or cell culture flasks. 

## 3. Biomedical Applications of Bio-Inspired Extreme Wetting Surfaces

Bio-inspired surfaces with extreme wetting properties can be directly applied to various biomedical applications, such as functional cell and tissue culture and analysis, biomedical devices, and lab-on-a-chip devices. This section reviews the recent developments in biomedical platforms using bio-inspired surfaces.

### 3.1. Cell Patterning for Cellular Interaction Studies

Protein adsorption and cell adhesion strongly depend on surface topology and chemistry. Cell interactions with superhydrophilic and superhydrophobic surfaces fabricated by patterning have been extensively investigated [15,16,111,112,113,114,115,116,117,118,119]. Piret *et al.* observed that Chinese Hamster Ovary K1 cells adhered selectively to micropatterned superhydrophilic regions while cell adhesion was suppressed on a superhydrophobic surface [16]. Similarly, Ishizaki *et al.* showed that surface physicochemical properties (e.g., roughness and wettability) affect cell adhesion and cell-cell interactions [14]. In particular, Mouse 3T3 fibroblast cells immediately adhered to the superhydrophilic regions after seeding, whereas the cells barely adhered to the superhydrophobic surfaces. This difference in cell attachment was attributed to the preference of protein absorption on superhydrophilic regions. Cultured cells selectively changed their shapes and adhesive directions depending on the distances between superhydrophilic regions. Moreover, cell-cell direct communication occurred between cells in neighboring spots below a patterned cell distance of 250 μm. 

The cultivation of multiple cell types in separated but adjacent compartments has proven crucial for mimicking and evaluating various biological processes, such as intercellular communication and cell signaling with respect to tissue and organism development. Efremov *et al.* introduced multiple cell types patterning on a hydrophilic porous polymer substrate bearing a patterned, superhydrophobic border [115]. These superhydrophobic border patterned hydrophilic substrates were created in two steps: First, a hydrophilic nanoporous polymer layer was formed by a UV polymerization reaction. Then, a hydrophobic photographing material was subsequently placed on top of the hydrophilic polymer layer. This impregnated layer was UV irradiated again using a photomask to produce the superhydrophobic borders. The resulting surface was used to investigate cell migration and signaling by pre-patterned cell co-cultivation in a mutual culture medium. The migration of MLTy-mCherry and HeLa-GFP cell lines across the thin superhydrophobic border was monitored for different geometries during three days of co-culture. The thin border prevented cell migration and can be used to precisely cultivate multiple cell types without intermingling. In parallel, the cell signaling mechanism was also investigated using Wnt proteins—a major signaling protein class emitted by organizing boundary zones. Specifically, they cultured Wnt ligand expressing zebrafish fibroblast Pac-2 cells adjacent to but without direct cell contact of cells between each pattern. The proteins propagated independently in the extracellular space of a tissue without direct cell-cell contact.

**Figure 8:**
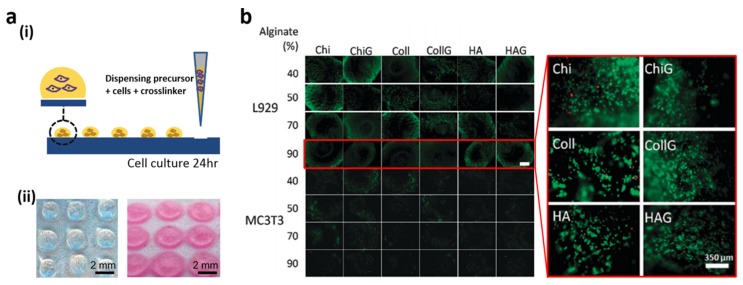
(**a**) (**i**) Three-dimensional (3D) hydrogel array on hydrophilic patterned spots; (**ii**) The images before and after 24 h of immersion in culture medium of alginate based 3D hydrogels; (**b**) Fluorescent microscopy images of live (green)/dead (red) cells in the 24 different hydrogels after 24 h of cell culture. Reproduced from [120] with permission by Royal Society of Chemistry, Copyright 2012.

Cell-cell and cell-biomaterial interactions have been widely investigated on flat 2D surfaces; however, studies in a 3D environment are more valuable because they mimic *in vivo* cell microenvironments better [121,122,123,124,125]. Salgado *et al.* investigated 3D interactions between cells and polymeric materials on hydrophilic patterned superhydrophobic substrates [120]. A superhydrophobic PS substrate was subjected to UV/ozone irradiation using a square-patterned photomask. Contrasting wettabilities enabled the deposition of various 3D hydrogel volumes on the hydrophilic patterns (Figure 8a(i)). 3D cell-hydrogel interactions were investigated for L929 fibroblast and MC3T3-E1 pre-osteoblast cell lines. A total of 24 different hydrogels were prepared by mixing alginate (Alg) with different ratios of chitosan (Chi), collagen (Coll), hyaluronic acid (HA), and gelatin (G). 5 μL of polymer/crosslinking solutions mixed with cells were deposited on the substrate and incubated for 24 h to analyze the cytocompatibility of 24 different hydrogel compositions (Figure 8a(ii)). After 24 h of cell culture, all the materials were analyzed in terms of cell viability, cell quantification, and cell metabolic activity assessment. Figure 8b shows fluorescent microscopy images of live (green) /dead (red) cells in the hydrogels after 24 h of culture. The number of dead cells was relatively low in an HA/G mixture containing 40% Alg, but high in the Coll mixture containing 40% Alg. They revealed that high and low viability of L929 fibroblast cells could be seen in 70% Alg content hydrogels containing Coll and HA, and hydrogels containing Chi, respectively. L929 cells exhibited high viability in Coll and HA-type hydrogels containing 70% Alg but poor compatibility with Chi-based hydrogels. In addition, the presence of Coll improved pre-osteoblast MC3T3-E1 cell viability because these fibers constitute the most abundant protein structure in pre-bone (osteoid) and bone native tissue. 

### 3.2. Functional Cell Spheroid Culture

3D cell culture environments facilitate a more effective study of cell-cell and cell-extracellular matrix (ECM) interactions. In particular, 3D cell spheroids provide more *in vivo* like microenvironments to the cells [126,127]. They have also attracted considerable attention because of their enhanced therapeutic functions compared with cells cultured on 2D substrates. Conventional dish culture, spinner flask culture, and hanging drop technique have been typically used to form 3D cell spheroids [128,129,130,131]. Among the methods, hanging drop culture benefits from cell size controllability and viability [132,133,134] and can be coupled with a hydrophilic patterned superhydrophobic surface acting as a culture platform. Lee *et al.* fabricated a wettability-patterned surface using a mussel-inspired adhesive polymer pDA by photolithography on a fluorosilane-coated superhydrophobic substrate [135]. Human mesenchymal stem cells (MSCs) and rat islet cells (ICs) were cultured on the pDA-patterned surface by the hanging drop method. 3D MSC-spheroids were successfully obtained on the surface and their vascular endothelial growth factor (VEGF) secretion increased to levels approximately 300% higher than the concentrations achieved by spinner flask culture. In addition, IC-spheroids exhibited approximately 200% sensitivity enhancement upon glucose stimulation. Spheroid cell size uniformity, viability, and functionality improved compared to cells cultivated by the hanging drop technique in conventional petri dishes. These results indicate that a spherical shape of the droplets improved cellular metabolic activity by enhancing cell-cell and cell-matrix interactions.

**Figure 9:**
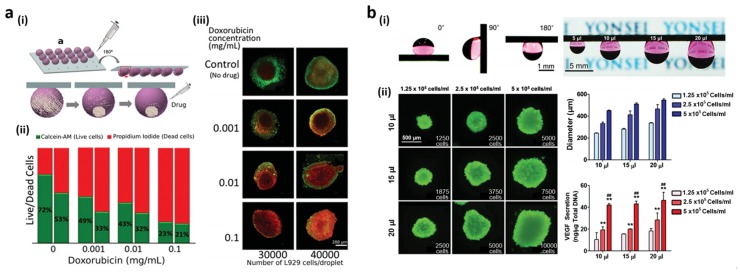
(**a**) (**i**) Hanging drop culture on the indentation-patterned superhydrophobic surface; (**ii**) Live (green)/dead (red) cell ratios 24 h after the addition of various doxorubicin concentrations for densities of 30,000 (left bar) and 40,000 (right bar) cells/droplet; (**iii**) Fluorescent images of L929 spheroids in the presence of doxorubicin (**ii**); (**b**) (**i**) Culture medium droplets adhered on the H_2_-exposed Pd-coated Si NWs for different tile angle (0°, 90°, and 180°) and medium volumes (5, 10, 15 and 20 μL); (**ii**) Live/dead cell staining, size distribution, and vascular endothelial growth factor (VEGF) protein secretion from spheroids after 4 days of culture at various cell densities and medium volumes. Reproduced from [18,136] with permission by John Wiley and Sons, Copyright 2014.

The usage of superhydrophobic surface without hydrophilic pattern has also proven useful for hanging drop spheroids culture. The 3D microenvironments on the superhydrophobic surface drastically reduce the volume of the cell culture media and prevent any interaction with the surface. Neto *el al*. used micro-morphology patterned superhydrophobic surface for spheroids culture [18]. They prepared a PS superhydrophobic surface and dented the surface under the applied force with sharp tips. Water droplets placed on the indentation exhibited high CAs but remained pinned because of the water penetration into the indentation. The maintained superhydrophobic properties lead to formation of spherical droplets on the surface while minimizing the contact areas between the droplet and surface. The formation of the 3D spheroids was achieved on the physically modified PS superhydrophobic surface (Figure 9a(i)). Cell-containing droplets remained suspended from the indentation-patterned surface even when the surface was turned upside down. A combinatorial analysis of the cell spheroids developed by gravitational force was performed using different cell densities or drug concentrations. Droplets containing immortalized mouse lung fibroblast cell line L929 with two densities of 30,000 and 40,000 cells/droplet were dispensed and cultivated on the inverted superhydrophobic surface. After 24 h of cell culture, these droplets were exposed to different amounts of doxorubicin anti-cancer drug to assess the dose-dependent response of the formed tumor spheroids. Figure 9a(ii) shows the drug screening results that were obtained from the stained fluorescent images (Figure 9a(iii)). Live (green)/dead (red) cell ratios decreased with increasing dose in both type of spheroids. Also, these ratios were higher at lower spheroid density. In addition, dead cells mostly accumulated in the spheroid inner regions (Figure 9a(iii)) because nutrients and waste release are more difficult to assess in larger and denser spheroids. 

To minimize cell-interface interactions during hanging drop spheroid culture, Seo *et al.* used superhydrophobic surface exhibiting reversible adhesion properties [136]. The gas-driven adhesion switchable superhydrophobic surface by deposition of hydrogen-sensitive Pd onto Si NW arrays is shown in Figure 7c,d. The adhesion switch properties of Pd-coated Si NW arrays directly applied to 3D spheroid formation using the hanging drop technique without any patterning process. Due to the adhesion properties of Pd-coated Si NW arrays after exposure of H_2_, various sizes of droplets containing human adipose-derived stem cell (hADSC) could be adhered right after the exposure to H_2_ and maintained under ambient air (Figure 9b(i)). Figure 9b(ii) shows the maintained viability of spheroids after four days of culture and controllability of spheroids size by different cell densities (1.25, 2.5, and 5.0 × 10^5^ cells/mL) and in differing medium volumes (10, 15, and 20 μL). It was shown that VEGF secretion from hADSC spheroids depended on spheroid size. hADSC spheroids displayed enhanced paracrine activity upon cell density and culture medium volume adjustment. Moreover, narrow size distribution and much enhanced VEGF secretion from hADSC spheroids were observed compared to those grown with conventional spinner flasks and hanging on petri dishes. This hanging drop approach improved hADSC viability, paracrine secretion, mitochondrial metabolic activity, apoptosis signaling, and ECM production compared to petri dish techniques. This enhanced cell functionality may stem from the reinforced cell-cell and cell-matrix interactions upon the formation of highly compact spheroids. Furthermore, the angiogenic potential of hADSC spheroids was evaluated as a functional assay. The conditioned medium obtained from hADSC spheroids cultured on Pd-coated Si NW arrays enhanced proliferation and accelerated the capillary formation of human endothelial cells.

### 3.3. Biomedical Devices

In biomedical applications for implanted medical devices, adherence of undesired biological matter on the surface has to be prevented. For example, an antibacterial property of the surface is crucial to prevent inflammation of the implanted device or contamination during cell/tissue culture process. To fabricate antibacterial surface, various methods have been developed [137,138,139,140,141,142,143,144,145,146]. In particular, a bio-inspired superhydrophobic surface has received attention as a potential antibacterial surface owing to its antifouling property, which can prevent bacterial adhesion onto the surface [147,148,149,150,151]. 

**Figure 10:**
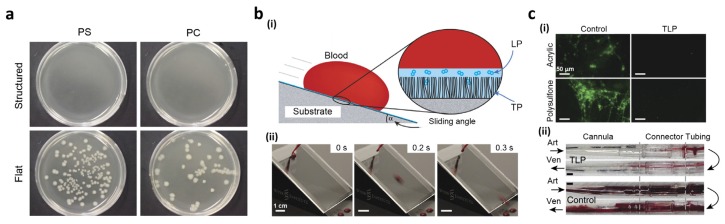
(**a**) The bacterial growth on the PS and polycarbonate (PC) structured and flat substrates; (**b**) (**i**) Blood repellency on slippery tethered-liquid perfluorocarbon (TLP)-coated surfaces; (**ii**) Photographs of a sliding blood droplet on the slippery surface; (**c**) (**i**) Fluorescent micrographs of fibrinogen on acrylic or polysulfone surfaces with or without TLP coating; (**ii**) Photographs of polyurethane cannulae, polycarbonate connectors, and PVC tubing with (top) or without (bottom) TLP coating after 8 h of blood flow. Reproduced from [148] under CC BY license. Reproduced from [152] with permission by Nature Publishing Group, Copyright 2014.

Privett *et al.* developed xerogel, comprising a mixture of silica colloids, fluoroalkoxysilane, and backbone silane [147]. Low surface energy and hierarchical structure by fluorinated silica nanoparticles allow superhydrophobic properties of the xerogel-coated surface. The antibacterial property of the xerogel was characterized using conventional flow cell assay with gram-positive *Staphylococcus aureus (S. aureus)* and gram-negative *Pseudomonas aeruginosa (P. aeruginosa*). The bacterial adhesions to the superhydrophobic xerogel-coated surface were significantly reduced by 99% and 98% compared with the blank surface, respectively. The result showed that the superhydrophobic surface could be applied to antibacterial applications; however, more practical fabrication methods with cost-effective and simple procedures need to be developed to use it as a versatile platform. Feschauf *et al.* reported antibacterial properties of superhydrophobic PS, polycarbonate (PC), and polyethylene (PE) surfaces using gram-negative *Escherichia coli* (*E. coli*) [148]. The superhydrophobic PS, PC, and PE surfaces were fabricated using a simple cast with a micro/nanostructured PDMS mold. To test the antibacterial properties of structured PS, PC, and PE surfaces, 10 μL of *E. coli* bacterial solution was cultured for 24 h on the surfaces (Figure 10a). After 24 h, superhydrophobic surfaces yielded less than 100 colony forming units (CFUs) for PS and PE, and no bacteria were grown on the PC substrate. Meanwhile, flat PS and PC had 100,100 CFUs and PE had 25,800 CFUs. These results indicate that superhydrophobic surfaces could effectively prevent bacterial adhesion to less than 0.1% compared with flat surfaces.

Liquid slippery surfaces also have a great potential to be used for various medical devices because of their wide-range of anti-fouling properties regarding various liquids and environmental stresses. They maintain repellency across a broad range of temperatures, pressure, surface tension, and other conditions [153]. Epstein *et al.* investigated the ability of a slippery surface to prevent biofilm attachment [154]. The bacteria are presented on a smooth slippery surface, and there is no ability to anchor to the mobile interface in contrast with solid interface. Regardless of the underlying porous solid structure, the slippery surface prevented diverse biofilm accumulation over a period exceeding one week. In addition, it reduced bacterial attachment by 96%–96.6% compared with common polyethylene glycol functionalized anti-fouling surfaces. 

Liquid slippery surface can be easily integrated into any arbitrary geometries such as pipes. Leslie *et al.* applied slippery properties to tubing and catheters of indwelling medical devices to reduce morbidity and mortality originating from thrombosis, which involves the blood component fibrinogen and platelets, and biofouling of the medical devices [152]. To create non-adhesive and anti-thrombogenic surfaces, they prepared a tethered perfluorocarbon (TP) layer on the tube surface and then coated it with a liquid perfluorodecalin (LP) (Figure 10b(i)). The thin mobile liquid layer allowed the tethered-liquid perfluorocarbon (TLP) surface to effectively repel liquids even when the surface was in contact with a flowing, immiscible fluid, such as blood. A fresh, whole human blood droplet almost immediately slid off the surface (Figure 10b(ii)). They confirmed that the TLP surface decreased fibrin adhesion and polymerization compared to uncoated acrylic and polysulfone surfaces (Figure 10c(i)). Moreover, the slippery surface also reduced platelet adhesion compared to uncoated surfaces. These results indicate that the slippery surface reduces fibrin polymerization and suppresses both adhesion and activation of plates. Anti-thrombogenic properties of the slippery surface were investigated *in vivo* using TLP-coated polyurethane cannulae, polycarbonate connectors and medical-grade polyvinyl chloride cardiopulmonary perfusion tubing was assembled into an arteriovenous shunt. Figure 10c(ii) shows the TLP-coated polymer tubes that decrease occlusive thrombosis compared to control tubing after 8 h of flow. Therefore, these remarkable anti-fouling properties may be exploited in various extracorporeal circuits and indwelling devices. 

### 3.4. Open-Channel, Droplet-Based Lab-on-a-Chip

Open-channel droplet-based microfluidic systems have several benefits compared with conventional closed-channel based microfluidic devices in terms of easy introduction of sample liquid, low sample and energy consumptions, and rapid chemical and biological reactions [155,156,157,158,159,160,161,162,163,164,165]. Manipulation of droplet motions is most important for the open-channel droplet-based microfluidic system. You *et al.* presented a droplet-based microfluidic system on a superhydrophobic porous oxide membrane [157]. To guide the water droplets, they introduced hydrophilic pDA microline patterns on the superhydrophobic surface. A square shape of pDA was also patterned at the center of the pDA microline for more complex manipulation of the droplets. A liquid droplet moved along the microlines by gravitational force and stopped on the square, which exhibited enough surface energy to capture the droplet. This immobilized droplet only moved downward upon the addition of a second droplet. This droplet mixing ability was applied to the synthesis of monodisperse gold nanoparticles and rapid structural changes in proteins.

**Figure 11:**
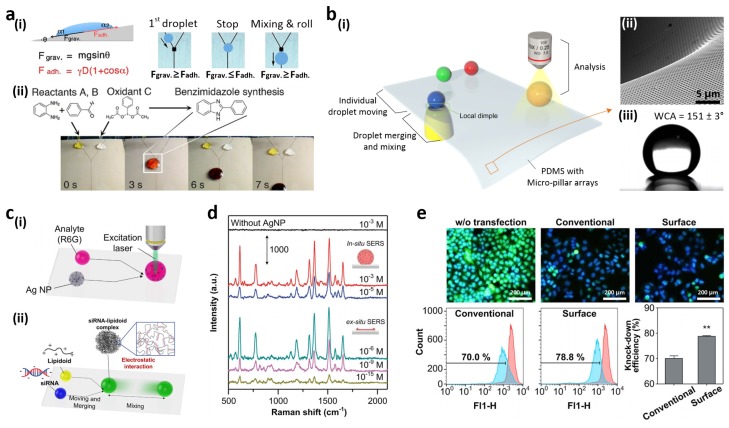
(**a**) (**i**) Droplet motion and mixing on a pDA micropatterned slippery device; (**ii**) Chemical reaction in organic solvent (THF) on the slippery device; (**b**) (**i**) Manipulation of water motions such as moving, mixing and analysis on a suspended PDMS substrate with micro-pillar arrays; (**ii**) SEM image of the dimple structure; (**iii**) Photograph of a water droplet on the PDMS substrate; (**c**) (**i**) Surface-enhanced Raman spectroscopy (SERS) measurement system; (**ii**) Small interfering RNA-lipidoid complex formation; (**d**) *in situ/ex situ* SERS analysis spectra at different concentrations of Rhodamine 6G (R6G) for R6G/Ag nanoparticle (NP) droplet mixture and an evaporated R6G/Ag NP droplet, respectively; (**e**) Fluorescent images and flow cytometry analyses of green fluorescent protein (GFP)-HeLa cells after transfection. Reproduced from [166,167] with permission by American Chemical Society, Copyright 2014, and permission by Nature Publishing Group, Copyright 2015.

However, hydrophilic patterned superhydrophobic surfaces cannot be used for organic solvent-based chemical and biological reactions and analyses. For a compatibility with various solvents, You *et al.* introduced a pDA micropatterned slippery surface [166]. To obtain a liquid guiding property, pDA microlines were patterned on a nanostructured surface before lubricant infiltration. The fabricated micropatterned slippery device was compatible with a variety of solvents such as water, ethanol, dimethyl sulfoxide, dimethylformamide, tetrahydrofuran (THF), n-hexane, 1,2-dichloroethane, acetic acid, 2-propanol, acetone, toluene, and diesel oil. Any solvents with surface tension greater than the lubricant (17.1 mN/m) were able to repel the infused lubricant located on top of the pDA microlines, and could be moved along the microlines by gravitational force. They introduced the square shape pattern for droplet mixing at the pDA microline intersection with the same mechanism as described above (Figure 11a(i)) [157]. Organic solvent-based chemical reactions were conducted on the pDA micropatterned slippery surface because of the wide solvent compatibility. An organic reaction between o-phenylenediamine and benzaldehyde was performed to produce 2-arylbenzimidazole. An 8 μL THF solution droplet containing two reactants and an 8 μL THF droplet containing an oxidant were deposited on the slippery surface (Figure 11a(ii)). The reaction yield (70.3%) increased compared to a typical bulk reaction using a 4 mL vial (52.1%) because of the rapid and homogeneous mixing. Moreover, the surface was cleaned and used repeatedly by simple washing with ultrasonication.

Patterned devices designed for droplet manipulation only provide limited control over fluidic operations, such as starting and stopping movement. Recently, Seo *et al.* produced a thin superhydrophobic PDMS substrate with micro-pillar arrays for path-programmable water droplet manipulations including droplet transportation, merging, mixing and analyses (Figure 11b(i)) [167]. When a vacuum pressure was applied at the bottom of a suspended PDMS substrate, the substrate was stretched and deflected downward to form a local dimple structure (Figure 11b(ii)). The micropillar array effectively reduced the contact area between the substrate and water droplets, resulting in superhydrophobicity (Figure 11b(iii)). By utilizing the dimple structure, water droplet motions could be individually controlled. Specifically, its border exhibited a positive curvature and the distance between adjacent pillars on the PDMS was greater than that on a flat substrate. The reduced number of micropillars lowered the water adhesion force, facilitating the detachment of the water droplet from the substrate. Therefore, the motions of water droplets on the substrate could not only be easily controlled by the vacuum-induced dimple structure, but also the moving path could be freely designed without additional patterning. The analytic performance of the substrate was examined by surface-enhanced Raman spectroscopy (SERS) (Figure 11c(i)). The *in situ/ex situ* SERS measurements were performed using Rhodamine 6G (R6G) with concentrations ranging from 10^−3^ to 10^−15^ M (Figure 11d). In *in situ* SERS measurements, individual water droplets containing R6G molecules and Ag nanoparticles (NPs) were transported and merged at the Raman detection spot before data collection. The detection limit of the R6G/Ag NP mixed droplet was 10^−5^ M due to freely diffusing R6G molecules and Ag NPs in the mixed droplet. To overcome this detection limit, the mixture was evaporated on the substrate and its components were concentrated within an area of hundreds of square micrometers. The R6G molecules were detected even at 10^−15^ M due to the accumulations of R6G molecules and Ag NPs for the *ex situ* SERS measurement. They also used the platform for generating uniform nanoparticle complexes for intracellular gene transfer. The lipidoid (ND98) complexes with green fluorescent protein-siRNA (siGFP) were formed by a simple merging and mixing process of the droplets (Figure 11c(ii)). Homogeneous mixing of the droplets could be achieved on the platform and the transfection efficiency of the lipidoid-siRNA complexes generated on the platform into GFP-HeLa cells (78.8% ± 0.5%) was increased compared to conventional manual pipetting mixing (70.0% ± 1.9%) (Figure 11e).

### 3.5. High-Throughput Cell Assay

High-throughput cell assays play an important role in screening the biological performance of various biomaterial compositions toward cellular behaviors, such as cell adhesion, viability, metabolic activity, and differentiation [168,169,170,171,172]. High-throughput cell assay using wettability-controlled surface enables simple, rapid, and low cost synthesis and analysis under different combinations of biomaterials even in a single experiment. 

**Figure 12:**
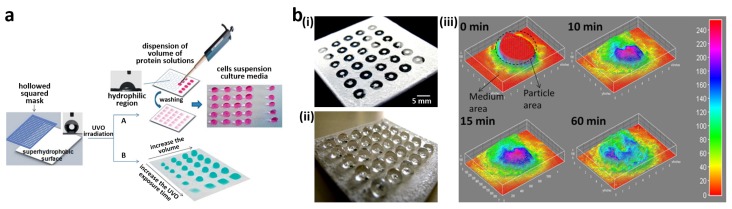
(**a**) High-throughput screening platform using a superhydrophobic surface patterned with hydrophilic spots; (**b**) (**i**) Superhydrophobic surface patterned with hydrophilic rings for high-throughput assay; (**ii**) Hydrogel- and phosphate-buffered saline (PBS) droplet-loaded substrate; (**iii**) Surface plots of release profiles of the fluorescein isothiocyanate labeled bovine serum albumin (BSA-FITC) from alginate hydrogels obtained by the acquisition of fluorescent microscopy images. Reproduced from [19,173] with permission by American Chemical Society, Copyright 2011, 2013.

Neto *et al.* fabricated a high-throughput screening platform using a hydrophilic spots patterned superhydrophobic surface by UV/ozone irradiation using a photomask (Figure 12a) [19]. Cell interactions with different mixtures of pre-absorbed proteins, albumin, and fibronectin were evaluated. Human serum albumin and human plasma fibronectin were individually dispensed with different relative amounts and protein concentrations on 20 hydrophilic spots. For the same total protein concentration, more cells were detected in spots treated with higher fibronectin content. This is in agreement with the passivating properties of albumin and the existence of integrin binding domains in fibronectin, which provide cell adhesiveness.

In addition to *in vitro* screening, high-throughput assays have emerged as attractive approaches to *in vivo* studies because of ethical issues and high costs related to animal testing. Recently, Oliveira *et al.* fabricated porous superhydrophobic scaffolds patterned with hydrophilic spots for high-throughput evaluation of foreign body response. The substrates with arrays of 36 combinations of biomaterials were used for subcutaneous implantation in Wistar rats. Lymphocyte and macrophage analyses were conducted along with a histological analysis of the surrounding tissue to assess the inflammatory response of distinct biomaterials. The response obtained on the hydrophilic patterned scaffolds was in agreement with the conventional implantation of the scaffolds, suggesting that the patterned scaffolds were useful for *in vivo* high-throughput assays involving few animals, short time, and low cost. 

However, the dispensing of small amount of droplets by handling is not suitable for a large-scale library of biomaterials in terms of dispensing rate and controllability. Ueda *et al.* presented superhydrophobic border patterned superhydrophilic surface for high-throughput cell assay [174]. The arrays of thousands of individually separated microdroplets were simply created on the substrate in a single step by dipping the substrate into an aqueous solution or rolling a droplet across the surface. Large volumes of molecules, particles, cells, or any other components in aqueous solutions were patterned in thousands of hydrophilic spots without manual pipetting or a liquid handling device. The superhydrophobic border patterned superhydrophilic surface was used in 3D hydrogel formation for cell screenings in a 3D microenvironment. Only several minutes were consumed to create an array of up to 85,000 hydrogels. Similarly, Oliveira *et al.* developed a superhydrophobic surface patterned with a ring-shaped hydrophilic region for high-throughput assays of bioactive agent delivery from a 3D hydrogel (Figure 12b(i)) [173]. They prepared aqueous solutions of 1%, 1.5%, and 2% of alginate and amounts of fluorescein isothiocyanate labeled bovine serum albumin (BSA-FITC) in concentrations of 0.1, 0.5, and 1 mg·mL^−1^, respectively. Protein release from alginate hydrogels encapsulating different BSA-FITC concentrations was investigated under nine sets of conditions. Hydrogel precursors mixed with BSA-FITC and a hydrogel crosslinker were sequentially dropped in each concentric superhydrophobic spot inside the ring-shaped hydrophilic region. After the crosslinking, physiological-like PBS was added dropwise to the rings, covering the cross-linked hydrogel. The high wettability contrast between the hydrophilic ring and superhydrophobic surroundings facilitated this deposition of PBS droplets, which retained their quasi-spherical shape (Figure 12b(ii)). BSA-FITC release profiles from alginate hydrogels was obtained by the acquisition of fluorescent microscopy images (Figure 12b(iii)). Acquired images showed overview of the distribution of BSA-FITC over time in the alginate hydrogels and PBS solution. The qualitative analysis of the distribution and amount of BSA-FITC was also performed through the linear relationship between the fluorescence intensities and BSA-FITC concentrations. The hydrogels with highest alginate concentration (2%) promoted slowest BSA-FITC release in PBS for all loading conditions due to tighter polymeric networks of the hydrogel. Also, highest BSA-FITC concentration (1 mg·mL^−1^) of the hydrogel led to fast release of BSA-FITC due to the high concentration gradient. 

## 4. Conclusions and Future Prospects

In this review article, we presented an overview of the design and fabrication of bio-inspired surfaces with extreme wetting properties and their applications for biomedical engineering. Principles governing these unique properties were described and representative living species ranging from the lotus leaf to the pitcher plant were reviewed with their bio-inspired surfaces. We also described the relevance of these properties in the biomedical field, such as cellular interactions on wettability patterned surfaces, 3D cell culture platforms for functional cell spheroids, and anti-fouling slippery surfaces for biomedical implantable devices. Furthermore, various functional approaches, such as droplet-based lab-on-chips and high-throughput cell assays, were introduced. 

Despite the efforts to apply bio-inspired surfaces to the biomedical field, they are still in the early stages compared with their conventional uses in other industries. There are some critical issues that need to be addressed for the wide usage of the bio-inspired surfaces as advanced biomedical platforms. For instance, surface durability and long-term stability of surfaces with extreme wetting properties should be improved. For *in vitro* cell/tissue culture, culture substrates should contain culture medium to deliver nutrients and bioactive molecules during the cell culture; however, the continuous contact between surface and culture medium leads to protein adsorption and the liquid penetration to micro/nanostructured surface, resulting in the change of surface wetting properties. Thus, the development of bio-inspired surfaces with long-term stability and durability may provide more *in vivo*-like microenvironments during cell culture and be used as an advanced platform to study cellular behaviors compared to conventional methodologies. Self-healing and stimuli-responsive composites that enable repairing damaged bio-inspired surfaces could be promising candidates for realization of the platform with long-term stability. Moreover, development of bio-inspired smart surfaces with the function of controllable capturing and releasing of biochemicals (e.g., drugs and cell-secreted proteins) can be used as an advanced platform for rapid and efficient disease diagnosis and drug discovery. Development and implementation of rapid and cost-effective fabrication methods is also desired; incorporating conventional methodologies for mass production such as spray coating, stamping, and 3D printing techniques they can be applied to fabricate bio-inspired surfaces. By addressing the aforementioned issues, these functional innovations can have a strong influence on the biomedical industry in the near future.
